# Epigenome-wide association study of leukocyte telomere length

**DOI:** 10.18632/aging.102230

**Published:** 2019-08-26

**Authors:** Yunsung Lee, Dianjianyi Sun, Anil P.S. Ori, Ake T. Lu, Anne Seeboth, Sarah E. Harris, Ian J. Deary, Riccardo E. Marioni, Mette Soerensen, Jonas Mengel-From, Jacob Hjelmborg, Kaare Christensen, James G. Wilson, Daniel Levy, Alex P. Reiner, Wei Chen, Shengxu Li, Jennifer R. Harris, Per Magnus, Abraham Aviv, Astanand Jugessur, Steve Horvath

**Affiliations:** 1 Department of Genetics and Bioinformatics, Norwegian Institute of Public Health, Oslo, Norway; 2 Department of Epidemiology and Biostatistics, School of Public Health, Peking University Health Science Center, Beijing, China; 3 Department of Epidemiology, Tulane University, New Orleans, LA 70118, USA; 4 Center for Neurobehavioral Genetics, Semel Institute for Neuroscience and Human Behavior, University of California Los Angeles, Los Angeles, CA 90095, USA; 5 Department of Human Genetics, David Geffen School of Medicine, University of California Los Angeles, Los Angeles, CA 90095, USA; 6 Centre for Genomic and Experimental Medicine, Institute of Genetics and Molecular Medicine, University of Edinburgh, Edinburgh, UK; 7 Centre for Cognitive Ageing and Cognitive Epidemiology, University of Edinburgh, Edinburgh, UK; 8 Department of Psychology, University of Edinburgh, Edinburgh, UK; 9 Epidemiology, Biostatistics and Biodemography, Department of Public Health, University of Southern Denmark, Odense C, Denmark; 10 Department of Clinical Genetics, Odense University Hospital, Odense C, Denmark; 11 Center for Individualized Medicine in Arterial Diseases, Department of Clinical Biochemistry and Pharmacology, Odense University Hospital, Odense C, Denmark; 12 Department of Physiology and Biophysics, University of Mississippi Medical Center, Jackson, MS 39216, USA; 13 Department of Cardiology, Beth Israel Deaconess Medical Center, Boston, MA 20892, USA; 14 The Framingham Heart Study, Framingham, MA 01702, USA; 15 Population Sciences Branch, Division of Intramural Research, National Heart, Lung, and Blood Institute, National Institutes of Health, Seattle, MD 20892, USA; 16 Public Health Sciences Division, Fred Hutchinson Cancer Research Center, Seattle, WA 98109, USA; 17 Children’s Minnesota Research Institute, Children’s Hospitals and Clinics of Minnesota, Minneapolis, MN 55404, USA; 18 Centre for Fertility and Health, Norwegian Institute of Public Health, Oslo, Norway; 19 Center of Development and Aging, New Jersey Medical School, Rutgers State University of New Jersey, Newark, NJ 07103, USA; 20 Department of Global Public Health and Primary Care, University of Bergen, Bergen, Norway; 21 Department of Biostatistics, Fielding School of Public Health, University of California Los Angeles, Los Angeles, CA 90095, USA

**Keywords:** DNA methylation, leukocyte telomere length, multi-ancestry

## Abstract

Telomere length is associated with age-related diseases and is highly heritable. It is unclear, however, to what extent epigenetic modifications are associated with leukocyte telomere length (LTL). In this study, we conducted a large-scale epigenome-wide association study (EWAS) of LTL using seven large cohorts (n=5,713) – the Framingham Heart Study, the Jackson Heart Study, the Women’s Health Initiative, the Bogalusa Heart Study, the Lothian Birth Cohorts of 1921 and 1936, and the Longitudinal Study of Aging Danish Twins. Our stratified analysis suggests that EWAS findings for women of African ancestry may be distinct from those of three other groups: males of African ancestry, and males and females of European ancestry. Using a meta-analysis framework, we identified DNA methylation (DNAm) levels at 823 CpG sites to be significantly associated (P<1E-7) with LTL after adjusting for age, sex, ethnicity, and imputed white blood cell counts. Functional enrichment analyses revealed that these CpG sites are near genes that play a role in circadian rhythm, blood coagulation, and wound healing. Weighted correlation network analysis identified four co-methylation modules associated with LTL, age, and blood cell counts. Overall, this study reveals highly significant relationships between two hallmarks of aging: telomere biology and epigenetic changes.

## INTRODUCTION

Telomeres are the (TTAGGG)_n_ repeats located at the ends of each chromosome. Their broad function is to prevent genomic instability [1]. Telomeres in adult germ cells [2], bone marrow [3, 4] and embryonic stem cells [5] are largely maintained by telomerase. After birth, however, telomeres in somatic cells gradually shorten because of the repressed activities of telomerase [3–6]. In cultured cells, when telomeres become critically short, the cell reaches replicative senescence [1, 7]. Telomere length (TL) is reported to be shorter in leukocytes of men than women, but this sex difference may depend on the measurement method [8]. In their meta-analysis of data from 36 cohorts with a total of 36,230 participants, Gardner and colleagues found longer telomeres in women only for the terminal restriction fragments (TRF) Southern blot method [8]. By contrast, no sex effect was detected for the other TL measurement methods including the widely used quantitative real-time polymerase chain reaction (qPCR) protocol originally described by Cawthon [9]. TL is also shorter in leukocytes of individuals of European ancestry than individuals of African ancestry [10, 11]. Further, leukocyte telomere length (LTL) is associated with the two disease categories that largely define longevity in contemporary humans—cancer and cardiovascular disease [12–14]

High heritability estimates for LTL have been reported irrespective of the methods used for measuring LTL; reported heritability estimates are between 36% and 82% based on Southern blot [15–18], and between 51% and 76% based on qPCR [19, 20]. Genome-wide association studies (GWAS) conducted in large observational cohorts have identified 11 loci associated with LTL [21–24]. A subset of these loci harbor telomere maintenance genes. These loci, however, explain only a small proportion of the genetic variance in LTL. Similarly, relatively little is known about epigenetic changes and LTL. Here, we focus on the relationship between LTL and DNA methylation levels in leukocytes. Epigenome-wide association studies (EWAS) have emerged as a powerful tool for evaluating genome-wide changes in DNAm for a given phenotype of interest [25]. Previous studies have explored the association between DNAm and LTL [26–28], but these studies were somewhat limited due to moderate sample sizes or the focus on specific regions in the genome. Here, we conduct the largest EWAS of LTL to date in different groups defined by sex and ethnicity.

## RESULTS

### Epigenome-wide association study of leukocyte telomere length

**Figure 1:**
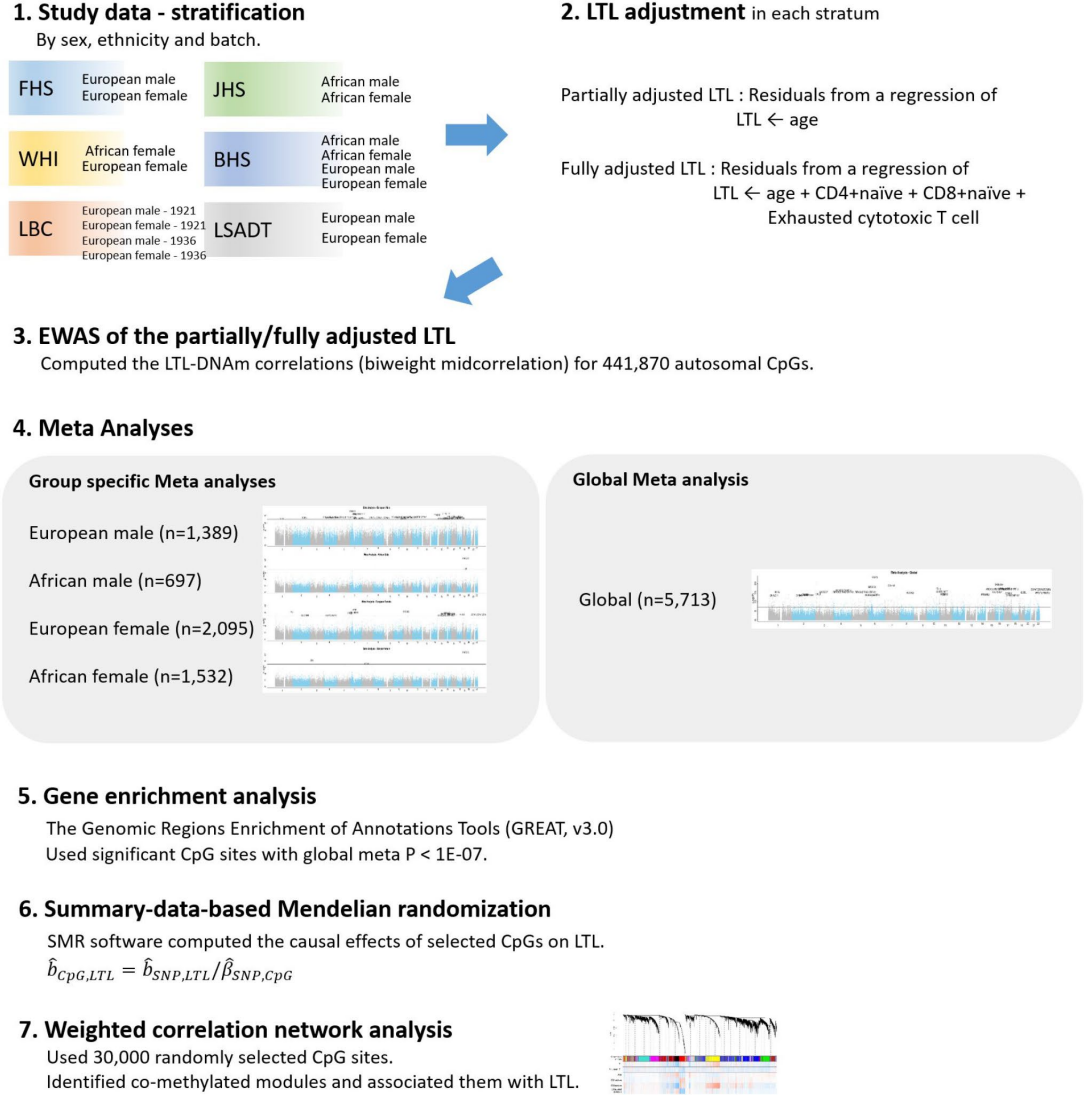
**Analysis flow chart.**

We considered two sets of adjustments for LTL confounders: 1) partially adjusted LTL for age, sex, and ethnicity and 2) fully adjusted LTL for age, sex, ethnicity, and imputed white blood cell counts (CD4+ naïve, CD8+ naïve and exhausted cytotoxic T cell). We conducted a large-scale multi-ancestry EWAS of the partially and fully adjusted LTL using seven cohorts – the Framingham Heart Study (FHS, n=874), the Jackson Heart Study (JHS, n=1,637), the Women’s Health Initiative (WHI, n=818), the Bogalusa Heart Study (BHS, n=831), the Lothian Birth Cohorts (LBC1921 and LBC1936, n=403 and n=906, respectively), and the Longitudinal Study of Aging Danish Twins (LSADT, n=244). The analysis flow is depicted in Figure 1. We note that adjustment in this script indicates a mixture of data stratification and regression adjustment.

Overall, 8,716 CpG sites were significantly (P<1E-07) associated with the partially adjusted LTL in the global meta-analysis. The top four genes with the largest number of significant CpGs were *VARS* (16 CpGs), *PRDM16* (15 CpGs), *MAGI2* (14 CpGs) and *MSI2* (13 CpGs). In the group-specific meta-analyses, we found 87 significant CpGs in men of European ancestry, 14 significant CpGs in men of African ancestry, 298 significant CpGs in women of European ancestry, and 20 significant CpGs in women of African ancestry (Supplementary File 1).

We identified 823 significant (P<1E-07) CpG sites associated with the fully adjusted LTL through the global meta-analysis. Our statistical significance threshold (1E-07) corresponds to a 5% family-wise error for 450K array studies [29]. Table 1 presents the top 30 CpGs among the 823 significant CpGs and groups them by their annotated gene names. Among the top 30 CpGs, six were in *VARS*, two were in *YPEL2* and two were in *XRCC3*. The CpGs highlighted by an asterisk in Table 2 were more strongly associated with LTL in one or two sex and ethnicity-specific groups than in the rest of the groups. Specifically, the LTL-DNAm correlations at cg27343900 (in *ERGIC1*) and cg12798040 (in *XRCC3*) were stronger in men of European ancestry than in women of African ancestry. The LTL-DNAm correlation at cg27106909 near *YPEL3* was stronger in men of European ancestry than in women of European ancestry.

**Table 1:** The top 30 most significant CpG sites associated with the fully adjusted LTL.

**CpG**	**Gene**	**Chr**	**Relation to UCSC CpG island**	**UCSC RefGene group**	**Meta-Analysis**				
					**Global meta Z (P) n=5,713**	**European male Z (P) n=1,389**	**African male Z (P) n=697**	**European female Z (P) n=2,095**	**African female Z (P) n=1,532**
cg08899667	*VARS*	6	N_Shelf	Body	-10.1 (4E-24)	-5.2 (3E-07)	-6.0 (2E-09)	-5.1 (4E-07)	-4.2 (3E-05)
cg02980249	*VARS*	6	N_Shelf	Body	-8.7 (2E-18)	-5.8 (5E-09)	-4.0 (6E-05)	-4.8 (2E-06)	-3.4 (7E-04)
cg02597894	*VARS*	6	N_Shelf	Body	-8.1 (4E-16)	-4.8 (2E-06)	-4.2 (3E-05)	-5.2 (2E-07)	-2.7 (6E-03)
cg04368724	*VARS*	6	N_Shelf	Body	-8.0 (9E-16)	-3.0 (2E-03)	-5.0 (5E-07)	-4.2 (3E-05)	-4.0 (8E-05)
cg04018738	*VARS*	6	N_Shelf	Body	-8.0 (2E-15)	-3.6 (3E-04)	-4.6 (4E-06)	-4.4 (1E-05)	-3.5 (4E-04)
cg24771152	*VARS*	6	N_Shelf	Body	-7.8 (6E-15)	-3.8 (2E-04)	-4.3 (2E-05)	-4.0 (6E-05)	-3.7 (2E-04)
cg20507228	*MAN2A2*	15	-	Body	-9.2 (5E-20)	-5.4 (8E-08)	-5.7 (2E-08)	-3.6 (3E-04)	-3.5 (4E-04)
cg08972170	*C7orf41*	7	-	Body	-9.0 (2E-19)	-3.7 (2E-04)	-4.9 (8E-07)	-4.1 (5E-05)	-5.4 (7E-08)
cg27343900*	*ERGIC1*	5	-	Body	-8.8 (1E-18)	-6.1 (8E-10)*	-5.1 (3E-07)	-4.2 (2E-05)	-2.4 (2E-02)
cg10549018	*TLL2*	10	-	Body	-8.6 (1E-17)	-5.3 (1E-07)	-3.9 (1E-04)	-4.5 (8E-06)	-4.0 (7E-05)
cg26709300*	*YPEL3*	16	N_Shore	1stExon;Body	-8.6 (1E-17)	-3.9 (8E-05)	-5.4 (6E-08)*	-2.4 (2E-02)	-4.8 (1E-06)
cg27106909*	*YPEL3*	16	N_Shore	1stExon;5′UTR;5′UTR	-8.5 (2E-17)	-5.6 (2E-08)*	-5.1 (3E-07)	-2.5 (1E-02)	-3.4 (6E-04)
cg12798040*	*XRCC3*	14	-	Body	-8.5 (2E-17)	-5.4 (8E-08)*	-5.4 (8E-08)*	-4.1 (4E-05)	-2.2 (2E-02)
cg02194129	*XRCC3*	14	-	Body	-8.3 (1E-16)	-4.9 (1E-06)	-5.0 (5E-07)	-4.3 (2E-05)	-2.6 (9E-03)
cg19841423*	*ZGPAT;LIME1*	20	S_Shore	Body;TSS1500	-8.4 (3E-17)	-5.0 (6E-07)	-5.5 (5E-08)*	-3.7 (2E-04)	-2.7 (8E-03)
cg02810967	*NCAPG;DCAF16*	4	S_Shore	Body;TSS1500	8.3 (9E-17)	4.4 (1E-05)	5.4 (9E-08)	4.1 (4E-05)	2.8 (5E-03)
cg19935065	*DNTT*	10	-	TSS1500	-8.1 (4E-16)	-3.5 (4E-04)	-4.9 (1E-06)	-5.0 (5E-07)	-3.2 (1E-03)
cg11093760	*CILP*	15	-	5′UTR;1stExon	-8.1 (5E-16)	-5.9 (4E-09)	-4.1 (5E-05)	-3.3 (1E-03)	-3.1 (2E-03)
cg19097500	*NFIA*	1	N_Shore	TSS1500	-8.1 (6E-16)	-5.4 (7E-08)	-3.7 (2E-04)	-3.7 (2E-04)	-3.6 (3E-04)
cg09626867	*EXOSC7*	3	-	Body	-8.1 (7E-16)	-5.2 (2E-07)	-4.1 (3E-05)	-4.5 (6E-06)	-2.8 (5E-03)
cg04509882	*EIF4G1*	3	-	Body;1stExon;5′UTR	-8.1 (8E-16)	-5.5 (4E-08)	-4.3 (2E-05)	-3.3 (1E-03)	-3.1 (2E-03)
cg23661483	*ILVBL*	19	S_Shelf	Body	-8.0 (9E-16)	-3.7 (2E-04)	-4.3 (2E-05)	-5.4 (7E-08)	-3.3 (1E-03)
cg01012082	*NCOA2*	8	-	3′UTR	-8.0 (1E-15)	-4.7 (3E-06)	-4.0 (7E-05)	-4.4 (1E-05)	-3.4 (8E-04)
cg21461082	*PRMT2*	21	Island	Body	8.0 (2E-15)	2.9 (4E-03)	4.4 (9E-06)	4.5 (6E-06)	4.4 (1E-05)
cg25921609	*MYH10*	17	N_Shore	Body	-7.9 (3E-15)	-5.2 (3E-07)	-3.6 (3E-04)	-4.5 (6E-06)	-3.1 (2E-03)
cg24420089*	*PTDSS2*	11	N_Shore	Body	-7.8 (8E-15)	-3.4 (7E-04)	-5.8 (7E-09)*	-2.3 (2E-02)	-3.5 (5E-04)
cg07414525	*CHL1*	3	-	Body	-7.8 (9E-15)	-3.5 (4E-04)	-3.0 (3E-03)	-3.5 (5E-04)	-5.8 (6E-09)
cg14817906	*CNNM4*	2	-	Body	-7.7 (1E-14)	-4.4 (1E-05)	-4.1 (4E-05)	-3.9 (8E-05)	-3.2 (1E-03)
cg04860432*	*PTGER2*	14	S_Shore	Body	-7.7 (2E-14)	-5.8 (7E-09)*	-4.3 (1E-05)	-2.3 (2E-02)	-2.7 (7E-03)
cg23570810	*IFITM1*	11	N_Shore	Body	7.7 (2E-14)	4.2 (3E-05)	4.2 (2E-05)	4.2 (2E-05)	3.0 (2E-03)

* The CpGs were more strongly associated with LTL in one or two sex and ethnicity specific groups than in the rest of the groups.

**Figure 2:**
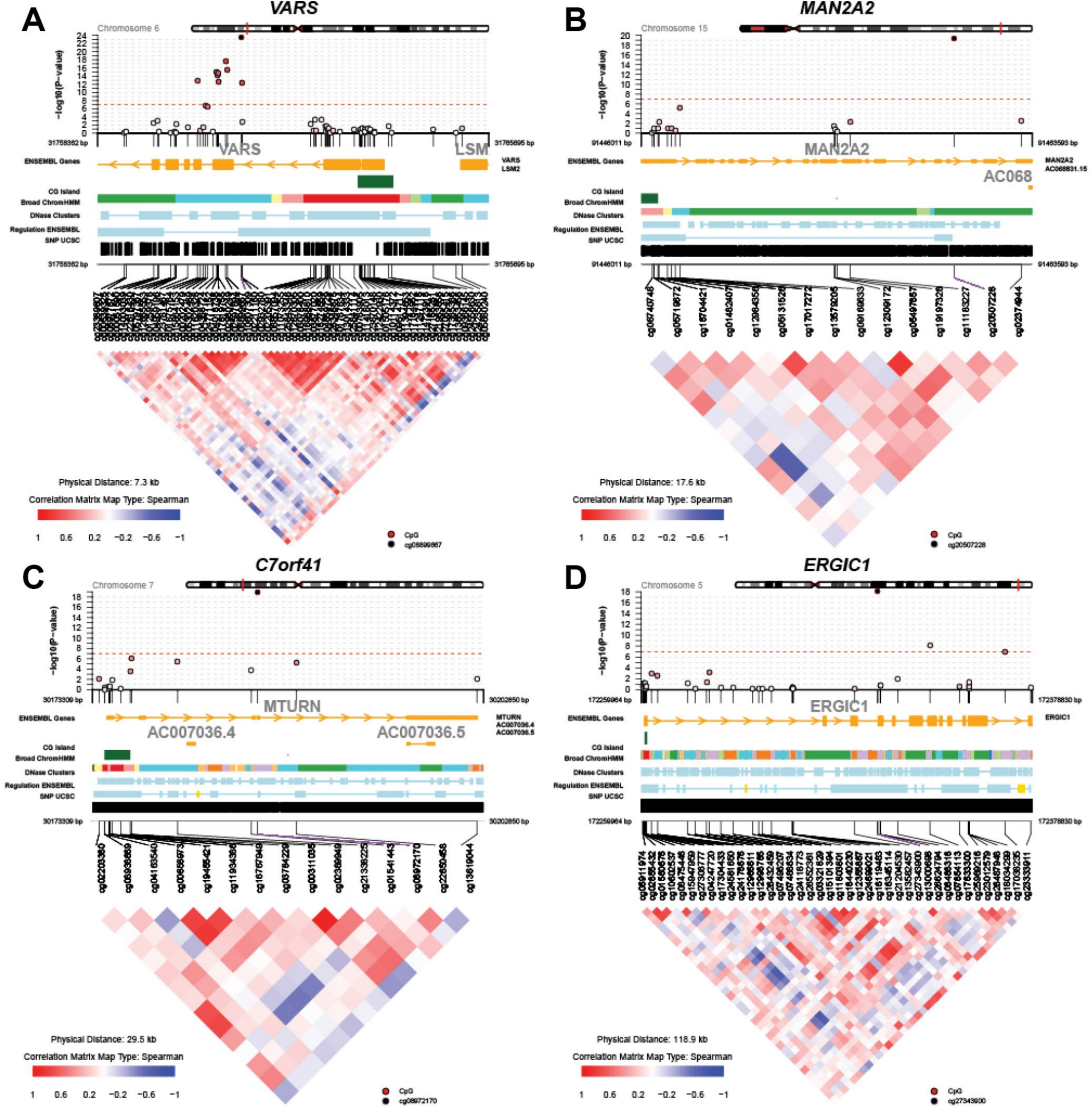
**Regional Manhattan plots and inter-CpG correlations for the top four genes identified in the global meta-analysis.** (**A**) *VARS*; (**B**) *MAN2A2*; (**C**) *C7orf41 (MTURN)*; (**D**) *ERGIC1*.

Figure 2 displays regional test statistics of LTL-associated CpGs on top of the local DNAm correlation structure for the top four genes listed in Table 1. *VARS* showed a cluster of CpGs above and right below the threshold of significance, while *MAN2A2*, *C7orf41* (current name, *MTURN*) and *ERGIC1* had one or two significant CpGs. The clusters detected in *VARS* might be because of the high probe density on the array and the strong inter-CpG correlations.

**Figure 3:**
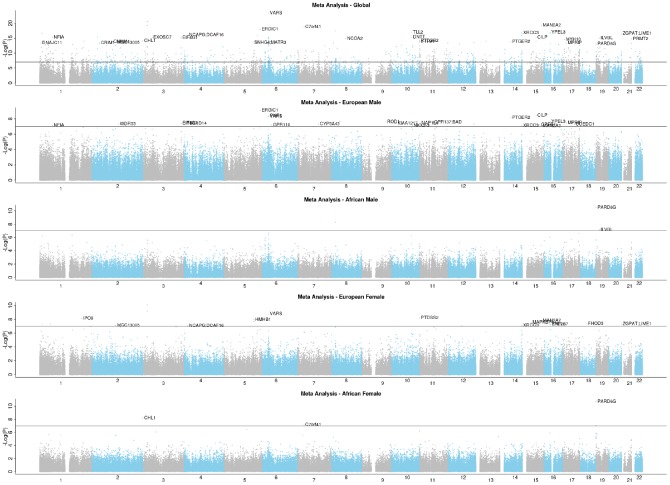
**EWAS Manhattan plots of the fully adjusted LTL.**

**Figure 4:**
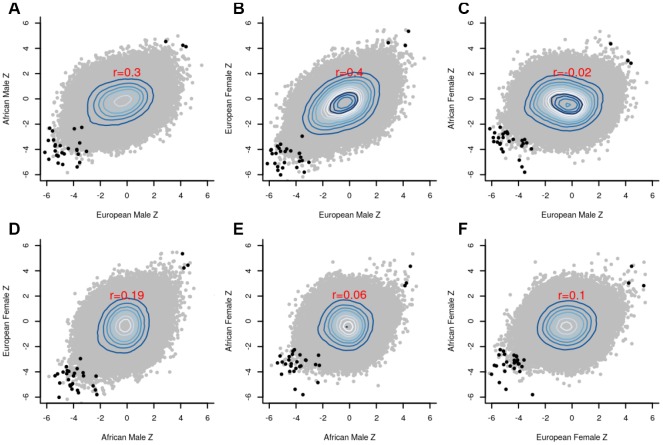
**Scatter plots between the group-specific meta-Z scores.** (**A**) European male *vs* African male; (**B**) European male *vs* European female; (**C**) European male *vs* African female; (**D**) African male *vs* European female; (**E**) African male *vs* African female; (**F**) African female *vs* European female; The black dots in the panels refer to the top 30 CpG sites detected by the global meta-analysis, whereas the grey dots indicate the remaining CpG sites. Pearson correlation coefficients (red font) reveal strong agreement (r=0.4) between males and females of European ancestry.

The group-specific meta-analyses also detected several significant (P<1E-07) CpGs associated with the fully adjusted LTL. Figure 3 shows that 25 CpGs were significant in men of European ancestry, three CpGs in men of African ancestry, 19 CpGs in women of European ancestry, and four CpGs in women of African ancestry. Figure 4 displays scatter plots across the four group-specific meta-analyses. The correlation coefficient of each scatter plot was lowest between African American females and European males (r=-0.02) and highest between European females and European males (r=0.40). Population and sample size differences between strata may influence the correlations. The black dots in the panels refer to the top 30 CpG sites detected through the global meta-analysis. Across the 30 CpGs, we did observe high correlations (r≈0.92).

### Functional enrichment analysis of LTL-associated CpG sites

To infer the biological meaning underlying LTL-associated CpG sites, the Genomic Regions Enrichment of Annotations Tool (GREAT) was used to associate differentially methylated probes (DMPs) with nearby genes of known pathway annotations. We performed both a gene-based and a region-based enrichment analysis for (1) all DMPs (n=850), (2) hypermethylated probes (n=95), and (3) hypomethylated probes (n=755). Analyzing all DMPs, we found 11 biological annotations to be significantly enriched with both the gene-based as well as the region-based test (Supplementary File 2, Figure S1, Table S1). Of these, five annotations showed a region-fold enrichment > 1.5; the circadian clock (3.9x), blood coagulation (1.9x), hemostasis (1.9x), wound healing (1.8x), and response to wounding (1.7x). Other annotations also related to circadian rhythm, blood coagulation and wound healing, further strengthening the main observations (Supplementary File 2, Tables S1, S2).

Next, analyzing hypomethylated probes only, we found that CpGs negatively correlated with LTL mainly explain the above-mentioned functional enrichment. In contrast, hypermethylated probes led to less significant enrichment p values, a finding likely due to the lower number of CpGs (Supplementary File 3). We observed an enrichment of genes involved in mitogen-activated protein kinase phosphatase activity and immune regulation (Supplementary File 2, Figure S1). As part of a robustness/sensitivity analysis, we repeated the enrichment study after excluding CpGs with single-nucleotide polymorphisms (SNPs) in the extension base (global minor allele frequency > 1%) or probes prone to mapping to multiple regions in the genome. Across overlapping annotations (n=1,590), we found high concordance with our initial findings (r=0.97, P<2.2E-16), indicating that our results are highly robust against potentially faulty probes. Details can be found in Supplementary File 3.

### DNA methylation in subtelomeric regions

We observed a higher proportion of the positive LTL-DNAm correlations in subtelomeric regions than in non-subtelomeric regions when we focused on the 823 significant CpGs that were associated with the fully adjusted LTL. The proportion of the positive LTL-DNAm correlations was 17.1% in the subtelomeric regions and 9.9% in the non-subtelomeric bodies (Chi-squared test, P=0.01; Supplementary File 2, Table S3). The subtelomeric regions were defined as each chromosome’s head and tail, each of which was 5% of each chromosome’s length. However, this approach may not be optimal for the following reasons: 1) the inter-CpG correlations may differ between the non-subtelomeric and subtelomeric regions; 2) one cannot clearly dichotomize genomic loci into non-subtelomeric and subtelomeric regions; and 3) the LTL measurements were not chromosome-specific but averaged across all chromosomes.

### Summary-data-based Mendelian randomization

We calculated the causal effects of the 823 CpGs (significantly associated with the fully adjusted LTL) on LTL using summary-data-based Mendelian randomization (SMR) [30] and found that 16 CpGs had a significant (P<0.05) causal effect on LTL (Supplementary File 2, Table S5). The causal effect of cg00622799 near *RTEL1* led to the lowest p-value (P= 6E-4) among the 823 CpGs when SNP rs909334 was used as an instrumental variable. A non-significant p-value (P=0.21) for the test for heterogeneity in independence instruments (HEIDI) is desirable because it indicates that rs909334 (instrumental variable) is the only SNP that influences LTL through the DNAm level at cg00622799. A GWAS of LTL [21] and cis methylation quantitative trait locus (cis-mQTL, a reduced GWAS of DNAm) [31] were used to obtain the SMR causal effects (betas), p-values and HEIDI p-values. The SMR p-value identifies possible methylation sites via which genetic variants (SNPs) might be influencing LTL. The HEIDI p-value then indicates the evidence that there is (1) a single causal SNP whose effect on LTL is mediated through the methylation CpG site (HEIDI P>0.05) or (2) different SNPs linked to the methylation level and LTL (HEIDI P<0.05).

Additionally, we examined whether the 823 CpGs overlapped significantly with 54,942 known cis-methylation QTLs. Strikingly, a highly significant number of CpGs (188 CpGs out of 823 CpGs) were known cis-mQTLs (hypergeometric test P= 1.02E-16). To carry out this overlap analysis, we retrieved 188 SNPs each of which corresponded to the 188 CpGs from the cis-mQTL summary statistics. Next, we looked up each of the 188 SNPs in the most recent GWAS catalogue database (v1.02, https://www.ebi.ac.uk/gwas/docs/file-downloads). 22 SNPs were associated with complex traits (Supplementary File 2, Table S6). Among these 22 SNPs, rs2540949 in *CEP68* was associated with atrial fibrillation, and rs17708984 in *TPM4* (GWAS P=6E-16) was associated with platelet count (Supplementary File 2, Table S6). Platelet count is related to blood coagulation and wound healing, which were identified through the functional gene enrichment analysis of the LTL-associated CpGs described above.

### Weighted correlation network analysis (WGCNA)

**Figure 5:**
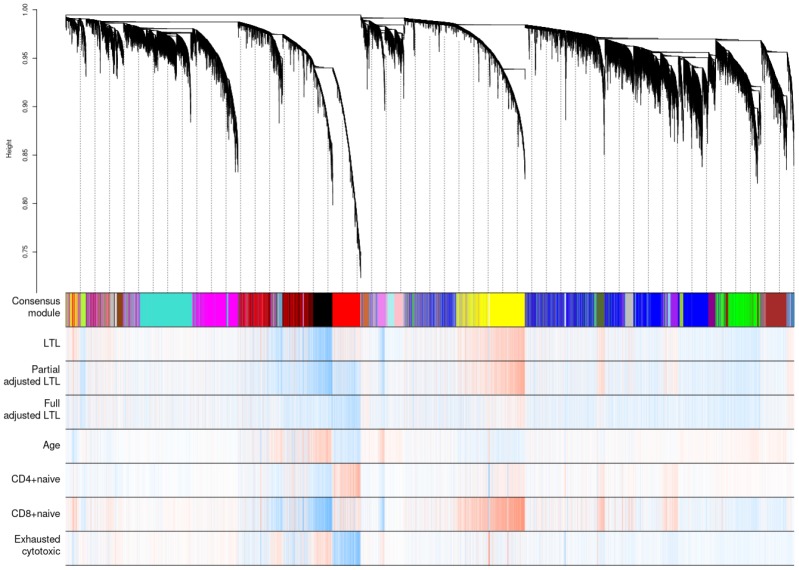
**Hierarchical clustering of CpG sites by weighted gene co-expression network analysis (WGCNA).** Each data point on the x-axis of the dendrogram refers to an individual CpG site. The color band ‘Consensus module’ displays co-methylated modules (clusters) in different colors. The other color bands highlight the degree of correlations between DNA methylation of CpG sites and traits of interest. Red represents a positive correlation, whereas blue represents a negative correlation.

**Figure 6:**
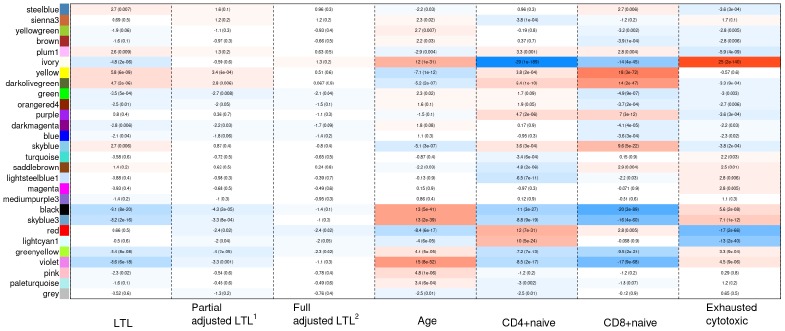
**Heat map of correlations between the co-methylated module representatives and LTL, the partially adjusted LTL, the fully adjusted LTL, age, and blood cell counts.** The numbers in the cells refer to meta-Z scores and their corresponding p-values. Meta-Z scores were calculated based on biweight midcorrelations between DNAm and a trait of interest in the six strata. ^1^Partially adjusted LTL for age, sex and ethnicity. ^2^Fully adjusted LTL for age, sex, ethnicity, CD4+ naïve, CD8+ naïve and exhausted cytotoxic T cell.

Weighted correlation network analysis (WGCNA) identified four important co-methylated modules (labeled black, red, ivory and yellow in Figure 5) using FHS, JHS and WHI (n=3,329). Hypermethylation in the black module was associated with increased age, shortened LTL, decreased CD8+ naïve T cell counts, and increased exhausted cytotoxic T cell counts, whereas hypermethylation in the red module showed opposite correlations. Elevated methylation levels in the yellow module were correlated with longer LTL and higher CD8+ naïve T cell counts. The ivory module had a pattern similar to the one in the black module. None of the modules revealed any strong correlation with the fully adjusted LTL, which is not surprising as this measure of LTL is adjusted for age and white blood cell type composition. The relationships between co-methylated module representatives and traits of interest (LTL, the partially adjusted LTL, fully adjusted LTL, age, and white blood cell counts) are displayed in Figure 6.

## DISCUSSION

This multi-ethnic EWAS of LTL is the largest to date and revealed strong associations between LTL and DNAm levels in all groups defined by sex and ancestry. Our stratified analysis showed that the EWAS findings for women of African ancestry are distinct from those of three other groups: males of African ancestry, males and females of European ancestry. A detailed analysis reveals that this difference does not reflect differences in sample size, age distribution, or LTL. We analyzed 1,532 blood samples from women of African ancestry, 697 from men of African ancestry, 1,389 from men of European ancestry, and 2,095 from women of European ancestry. Although men of African ancestry had the smallest sample size, their EWAS results were consistent with those from the two European groups.

Our unadjusted meta-analysis across the groups revealed profound relationships between TL and global DNA methylation levels, which largely reflect confounding by blood cell composition. However, one can observe genome-wide significant relationships between methylation levels and LTL even after adjusting for differences in blood cell composition. In particular, we report 823 CpGs (close to or within 557 genes) that are significantly correlated with the fully adjusted LTL. More than 88 percent (730 CpGs) of these 823 significant CpG sites exhibit a negative correlation with LTL, meaning that higher methylation levels are associated with shorter LTL at these CpG sites.

Among the 823 CpGs, the top 10 CpGs were linked to seven genes/loci (*VARS, MAN2A2, C7orf41, ERGIC1, TLL2, YPEL3 and XRCC3*). *VARS* encodes the enzyme Valyl-tRNA synthetase that is critical in eukaryotic translation [32]. Mutations in *VARS* cause neurodevelopmental disorders, such as microcephaly, cortical dysgenesis, seizures, and progressive cerebral atrophy [32, 33]. *MAN2A2* encodes alpha-mannosidase 2x that is active in N-glycan biosynthesis [34]. *MAN2A2* null males were largely infertile in mouse studies [35]. *C7orf41* (current official name, *MTURN*), encodes Maturin, a protein that controls neurogenesis in the early nervous systems [36]. *ERGIC1* encodes a cycling membrane protein that contributes to membrane trafficking and selective cargo transport between intermediate compartments [37, 38]. *TLL2* encodes Tolloid-like protein 2 [39] and is associated with attention-deficit/hyperactivity disorder [40]. *YPEL3* codes for Yippee-like 3, a protein that suppresses tumor growth, proliferation and metastasis in several types of cancer [41, 42]. *XRCC3* encodes a RecA/Rad51-related protein that maintains chromosome stability and repairs DNA damage [43, 44].

Functional enrichment studies demonstrate that the significant CpG sites were located near genes that play a role in circadian clock, blood coagulation, and wound healing, respectively. A rich literature links TL to circadian rhythm. For example, cellular senescence impairs circadian rhythmicity both in vitro and in vivo [45]. Sleep disorders and shorter sleep duration are associated with shorter telomeres [46, 47]. Telomerase and TERT mRNA expression are furthermore under the control of CLOCK-BMAL1 regulation (a core component of the circadian clock) and exhibit endogenous circadian rhythms [48]. CLOCK-deficient mice display shortened TL and abnormal oscillations of telomerase activity [48]. Our results are in line with these findings and support a relationship between LTL and circadian rhythm.

TL has also been associated with wound healing and blood coagulation. For example, mice with longer telomeres show higher wound healing rates of the skin [49]. Furthermore, exogenous delivery of the human TERT gene significantly improved wound healing in an aged rabbit model [50]. In humans, poor wound healing has been reported in individuals with dyskeratosis congenita, a rare congenital disorder caused by a defect in telomere maintenance [51]. While assigning causality remains a challenge, our findings do provide evidence that telomere functioning is associated with the circadian clock, wound healing and blood coagulation through the DNA methylome in a population-based sample. Future work is needed to further understand the mechanisms by which this is regulated and how it impacts human health and diseases.

Our findings were based on a considerably larger sample size (n=5,713) than previous studies. Buxton et al. (2014) used 24 blood and 36 Epstein-Barr virus cell-line samples of 44 to 45 years old males and identified 65 and 36 TL-associated gene promoters, respectively [27]. Gadalla et al. (2012) was based on a sample of 40 cases with dyskeratosis congenita and 51 controls [28], and the authors reported a positive correlation between LTL and methylation at LINE-1 and subtelomeric sites only among the cases. Bell and colleagues performed an EWAS of age, TL and other age-related phenotypes using 172 samples of female twins [26]. Due to the small sample size, the authors could not find genome-wide significant associations between DNAm levels and TL.

We adjusted LTL for imputed blood cell composition in addition to age, sex, and ethnicity, because blood cell composition confounds the relationship between DNAm [52, 53] and LTL [54]. Consistent with previous findings, our WGCNA analyses in Figure 5 also showed that the black, red, and yellow modules were highly related to both blood cell counts and LTL. One concern was that blood cell counts might be causally influenced by DNAm and LTL (i.e., blood cell counts might be a collider between DNAm and LTL), which may introduce bias in LTL-DNAm correlations. Thus, we ran another EWAS without considering blood cell counts and compared LTL-DNAm correlations before and after adjustment for blood cell counts (Supplementary File 1). The correlations listed in Table 1 became slightly weaker after adjustment for blood cell counts but remained significant nonetheless. However, the number of associated CpG sites was greatly reduced after adjustment for blood cell counts. Cell type heterogeneity is thus an important variable to consider in studies of telomere length. Future work should be extended to cell type-specific analysis as well as to tissues beyond whole blood.

We did not adjust LTL for cigarette smoking in our main analyses because smoking had a non-significant effect on LTL (FHS: P=0.83 for never *vs* former smoker and P=0.76 for never *vs* current smoker; WHI: P=0.20 for never *vs* former smoker and P=0.24 for never *vs* current smoker), though suggestive associations could be found in JHS (P=0.08 for never *vs* former smoker and P=0.02 for never *vs* current smoker). These results pointing to a very weak effect of smoking are consistent with those from Astuti and colleagues [55] who reported that 50 of 84 studies found no association between smoking and TL, although their meta-analysis concluded that smokers may have shorter TL. Our sensitivity analyses also revealed that all the 823 CpGs remained significant regardless of smoking variables. Our EWAS summary statistics includes this sensitivity analysis with additional adjustment for smoking (see the names of columns starting with “aaa_” in Supplementary File 1).

One limitation of our study is that it does not elucidate the biological pathways or mechanisms linking DNAm and LTL. In other words, our findings do not explain whether DNAm shortens or lengthens LTL, or whether LTL regulates DNAm. Second, we did not include genotypic information in our analyses. Other studies have suggested that genomic variants might regulate DNAm [31] and LTL [21–24, 56]. Third, LTL measurement is sensitive to the methods used for DNA extraction and LTL estimation [57]. Fourth, we only used EWAS and WGCNA to analyze the data. A supervised machine-learning approach for predicting TL based on DNAm levels will be described in a separate article [58].

This study represents the largest EWAS analysis of DNA methylation and LTL to date. We identified over 800 genome-wide significant CpG sites that are located in or near genes with links to circadian rhythm, blood coagulation and wound healing. These findings link two hallmarks of aging: epigenetic changes and telomere biology.

## MATERIALS AND METHODS

### Study population

The FHS Offspring Cohort started in 1971 to inaugurate epidemiological studies of young adults in Framingham, Massachusetts, USA. The FHS recruited 5,124 individuals and invited them to examinations at the FHS facilities [59]. The JHS recruited 5,306 African Americans from 2000 to 2004 in the Jackson metropolitan area, Mississippi, USA, to investigate risk factors for cardiovascular disease [60]. Participants provided medical history, social records and whole-blood samples. The WHI started in 1992 and enrolled 64,500 postmenopausal women aged between 50 and 79 years into either clinical trials or observational studies [61]. Among many sub-studies, WHI “Broad Agency Award 23” has provided both blood-based LTL and DNAm array data. The BHS started in 1972 and has recruited multiple waves of participants from childhood, adolescence and adulthood in Louisiana, USA [62]. The LBC1921 and LBC1936 are longitudinal studies of 550 individuals born in Scotland in 1921 and of 1091 individuals born in Scotland in 1936. The studies were set up in 1999 and 2004, respectively, with the aim of studying cognitive aging [63, 64]. The LSADT was initiated in 1995 and is a cohort-sequential study of Danish twins aged 70 years or more [65, 66]. Surviving twins were surveyed every second year until 2005. In 1997, whole blood samples were collected from 689 same-sex twins and the present study included all twin pairs who participated in the 1997 wave and for whom LTL measurements were available.

The sample size of each cohort used in this study as follows: FHS (n=874), JHS (n=1,637), WHI (n=818), BHS (n=831), LBC1921 (n=403), LBC1936 (n=906), and LSADT (n=244).

### Measurement of LTL

LTL was measured by either of two methods: Southern blot [67] or qPCR [9]. All cohorts used Southern blot, except for LBC1921 and LBC1936 that used qPCR. LTL measurement by Southern blot provides the mean of TRFs, whereas qPCR provides the ratio of telomeric template to glyceraldehyde 3-phosphate dehydrogenase. The average inter-assay coefficients of variation were 2.4% in FHS, 2.0% in JHS, 2.0% in WHI, 1.4% in BHS, 5.1% in LBC (LBC1921 and LBC1936 combined), and 2.5% in LSADT. Further details on the measurement of LTL in each cohort are provided in Supplementary File 2.

### Measurement of DNA methylation

DNAm data were generated on either of two different Illumina array platforms: the Illumina Infinium HumanMehtylation450 Bead-Chip (Illumina, San Diego, CA, USA) or the Illumina Infinium MethylationEPIC Bead-Chip (Illumina, San Diego, CA, USA). Beta values were computed, which quantify methylation levels between 0 and 1, with 0 being unmethylated and 1 being fully methylated. Further details on normalization and quality control of the data can be found in Supplementary File 2.

### Statistical analysis

We stratified the seven cohorts (FHS, JHS, WHI, BHS, LBC1921, LBC1936 and LSADT) by sex, ethnicity and batch, which resulted in 16 strata (Table 2).

**Table 2:** Sample size of the 16 strata used in the meta-analyses.

**Cohort**	**Stratum**	**Sample size**	**Mean age (range)**	**Mean LTL^2^ (range)**	**Age-LTL correlation^3^**
FHS	European female	442	57 (33-81)	7.07 (5.51-8.7)	-0.29
	European male	432	58 (36-82)	6.92 (5.59-8.52)	-0.34
JHS	African female	1034	56 (23-92)	7.22 (4.93-10.03)	-0.39
	African male	603	55 (22-93)	7.06 (5.12-9.24)	-0.45
WHI	African female	342	63 (50-80)	7.12 (5.57-9.06)	-0.24
	European female	476	68 (51-80)	6.77 (5.24-8.49)	-0.27
BHS	African female	156	44 (30-54)	7.34 (5.35-9.22)	-0.08
	African male	94	44 (33-49)	7.21 (5.60-9.47)	-0.17
	European female	315	43 (29-55)	6.82 (5.02-9.17)	-0.08
	European male	266	43 (28-52)	6.75 (5.27-8.54)	-0.18
LBC1921^1^	European female	242	79 (78-80)	3.99 (3.00-4.72)	-0.29
	European male	161	79 (78-81)	4.26 (3.46-5.31)	-0.29
LBC1936^1^	European female	448	70 (68-71)	4.05 (2.69-6.00)	0.01
	European male	458	70 (68-71)	4.33 (2.99-7.12)	0.17
LSADT	European female	172	79 (73-90)	5.79 (3.94-7.38)	-0.25
	European male	72	79 (74-87)	5.60 (4.53-6.78)	-0.17

^1^ LBC recruited adults living in and around Edinburgh and who were born in 1921 and 1936.

^2^ In kilobases; LTL measurement in TRF (Southern blot): FHS, JHS, WHI, BHS and LSADT; LTL measurement in T/S (qPCR): LBC1921 and LBC1936.

^3^ Pearson correlation coefficients.

In each of the 16 strata, we applied two sets of adjustments on LTL using a regression: 1) partially adjusted for age alone, and 2) fully adjusted for age and DNAm-based estimated cell type proportions (CD4+ naïve, CD8+ naïve T cell and exhausted cytotoxic T cell). In FHS and LSADT, we used a linear mixed model to regress LTL on the adjusting variable(s) (fixed effect) and family structure (random effect). In JHS, WHI, BHS, LBC1921 and LBC1936, an ordinary linear regression was used. The blood cell type proportions were estimated using Horvath’s DNAm age calculator (https://dnamage.genetics.ucla.edu/home), with the exception of LSADT where the blood cell counts were estimated using Houseman et al. (2012)’s method [68].

The ***R*** package for weighted gene co-expression network analysis (WGCNA; [69]) was used to compute epigenome-wide biweight midcorrelations between DNAm levels and adjusted LTL in each of the 16 strata. The biweight midcorrelation is an attractive method for computing correlation coefficients because 1) it is more robust than Pearson correlation and 2) unlike the Spearman correlation, it preserves the biological signal as shown in large empirical studies [70]. We focused on 441,870 autosomal probes that were shared between the 450K and the EPIC array. We combined the 16 sets of EWAS summary statistics into four group-specific or one global meta summary statistics as described in Figure 1. Meta Z values and the corresponding p-values were computed as $\sum Z_{i}w_{i}\text{/}\sqrt{\sum w_{i}^{2}}$ and $2\left(1-\Phi (|{\text{Z}}_{\text{meta}}|)\right)$, where *w_i_* is the square root of the sample size in the *i*th stratum, respectively.

Genomic Regions Enrichment of Annotations Tools (GREAT, v3.0) was used to predict the biological function of DMPs by associating both proximal and distal genomic CpG sites with their putative target genes [71]. GREAT implements both a gene-based test and a region-based test using the hypergeometric and binomial test, respectively, to assess enrichment of genomic regions in biological annotations. DMPs were uploaded to the GREAT web portal (http://great.stanford.edu/public/html/) and analyses were run using the hg19 reference annotation and the whole genome as background. Genomic regions were assigned to genes if they are between 5 Kb upstream and 1 Kb downstream of the TSS, plus up to 1 Mb distal. Pathway annotations from GO Biological Processes, GO Cellular Component, GO Molecular Function, MSigDB, and PANTHER were used to infer the biological meanings behind the DMPs that were associated with LTL. GREAT outputs statistics of the gene-based and region-based tests, which were subsequently adjusted for multiple testing using the Bonferroni correction.

The SMR executable software (https://cnsgenomics.com/software/smr/#Download) was used to calculate the causal effects of the selected CpGs on LTL [30]. The SMR obtains a causal effect estimate $\left({\hat{b}}_{CpG,LTL}={\hat{b}}_{SNP,LTL}\text{/}{\hat{\beta }}_{SNP,CpG}\right)$ by dividing the effect of a SNP on LTL $\left({\hat{b}}_{SNP,LTL}\right)$ by the effect of a SNP on CpG $\left({\hat{\beta }}_{SNP,CpG}\right)$. GWAS of LTL summary data by Codd and colleagues [21] was downloaded from the European Network for Genetic and Genomic Epidemiology consortium (https://downloads.lcbru.le.ac.uk/engage). The mQTL data by McRae and colleagues [31] were downloaded from the SMR website (http://cnsgenomics.com/data/SMR/LBC_BSGS_meta.tar.gz).

WGCNA performed a consensus network analysis using FHS, JHS and WHI. 30,000 randomly selected CpG sites were used to improve readability (resulting in a single cluster tree) and offset computational limitations. WGCNA hierarchically clustered the 30,000 CpGs based on their similarities. The merging threshold of clusters (modules) was 0.15. All the statistical analyses were performed using ***R*** version 3.5.1.

## Supplementary Material

Supplementary File 1

Supplementary File 2

Supplementary File 3
