# Supplementary material for: Epigenome-wide association study of leukocyte telomere length
Source: Aging (Albany NY). 2019 Aug 26;11(16):5876–94. doi: 10.18632/aging.102230 (PMC6738430; doi:10.18632/aging.102230)
Supplement: Supplementary File 2 [file aging-11-102230-s002.pdf]

SUPPLEMENTARY FILE 2

Functional enrichment analysis

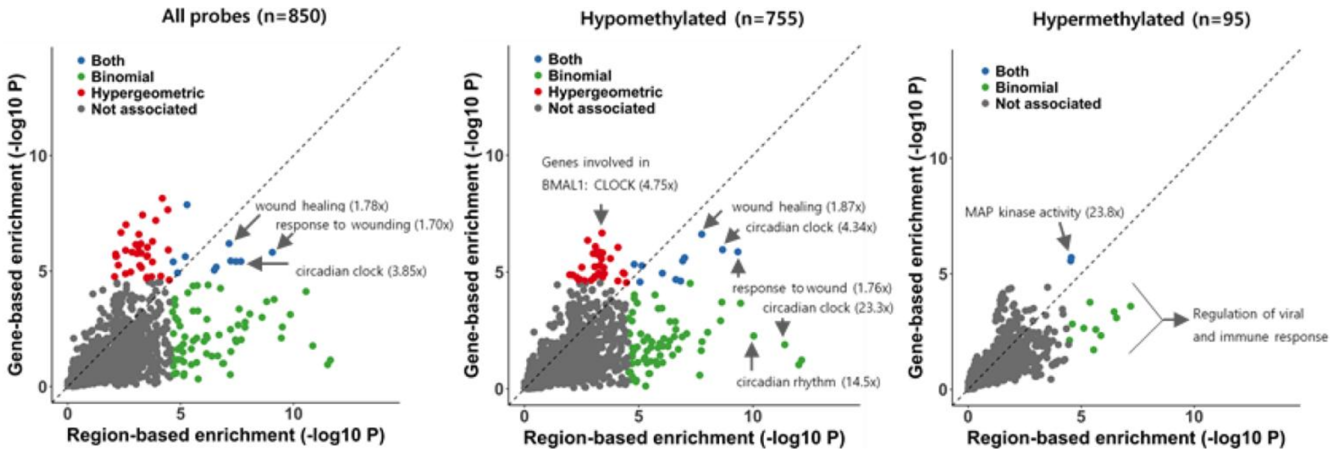

Supplementary Figure 1. Functional enrichment analysis highlights specific biological pathways associated with LTL-associated probes.

Differentially methylated probes (DMPs) were tested for enrichment of biological annotations using both a gene-based (hypergeometric) and a region-based (binomial) test. The x-axis shows the -log10 p-value of the binomial test, while the y-axis shows the association strength of the hypergeometric test. Annotations significant after Bonferroni correction are color-coded as follows: P<0.05 in both tests (blue), P<0.05 in the hypergeometric test only (red), P<0.05 in the binomial test only (green), P > 0.05 in both tests (grey). The annotations significant in both test and having the largest region fold enrichment are labeled in the graph. Results are shown for all probes (left), hypomethylated probes (middle), and hypermethylated probes (right).

Supplementary Table 1. Functional enrichment analysis highlights circadian rhythm annotations.

Ontology	ID	Description	Binomial p-value	Region Enrichment	Hypergeometric p-value	Gene Enrichment
MSigDB Pathway	REACTOME_CIRCADIAN_CLOCK	Genes involved in Circadian Clock	2.12E-09	4.34	1.1E-06	4.60
PANTHER Pathway	P00015	Circadian clock system	4.03E-12	23.26	1.3E-02	5.69
MSigDB Pathway	KEGG_CIRCADIAN_RHYTHM_MAMMAL	Circadian rhythm - mammal	9.21E-11	14.51	5.4E-03	5.26
GO Biological Process	GO:0032922	circadian regulation of gene expression	2.72E-05	6.94	2E-01	2.44
MSigDB Pathway	REACTOME_BMAL1_CLOCK_NPAS2_ACTIVATES_CIRCADIAN_EXPRESSION	Genes involved in BMAL1:CLOCK/NPAS2 Activates Circadian Expression	3.46E-05	3.55	2.8E-05	4.75
MSigDB Pathway	PID_CIRCADIANPATHWAY	Circadian rhythm pathway	1.91E-04	6.08	6.3E-02	3.20
GO Biological Process	GO:0042752	regulation of circadian rhythm	2.23E-04	4.52	3.2E-01	1.51
GO Biological Process	GO:0007623	circadian rhythm	2.52E-03	2.34	7.6E-02	1.80

Shown are results of the region-based (binomial) and gene-based (hypergeometric) functional enrichment test for all circadian rhythm related annotations tested using the GREAT method [1]. Differentially methylated probes (DMPs) were used as input and show a consistent signal of enrichment for LTL-associated CpG sites.

Supplementary Table 2. Functional enrichment analysis highlights blood coagulation and wound healing annotations.

Ontology	ID	Description	Binomial p-value	Region Enrichment	Hypergeometric p-value	Gene Enrichment
GO Biological Process	GO:0009611	response to wounding	4.6E-10	1.76	1.4E-06	1.63
GO Biological Process	GO:0042060	wound healing	1.7E-08	1.87	2.4E-07	1.90
GO Biological Process	GO:0007596	blood coagulation	9.8E-08	1.96	2.5E-06	1.91
GO Biological Process	GO:0007599	hemostasis	1.2E-07	1.95	3.3E-06	1.89
MSigDB Pathway	REACTOME_HEMOSTASIS	Genes involved in Hemostasis	9.2E-07	1.94	1.1E-05	1.89
GO Biological Process	GO:0061041	regulation of wound healing	7.5E-05	2.65	2.2E-04	2.85
GO Biological Process	GO:0060055	angiogenesis involved in wound healing	3.7E-03	6.48	1.1E-01	3.42
GO Biological Process	GO:0030193	regulation of blood coagulation	4.6E-05	3.29	1.3E-03	2.89
GO Biological Process	GO:0050818	regulation of coagulation	5.4E-05	3.11	7.9E-04	2.89
GO Biological Process	GO:0030195	negative regulation of blood coagulation	5.8E-05	5.42	1.7E-02	2.85
GO Biological Process	GO:0050819	negative regulation of coagulation	9.2E-05	4.58	7.9E-03	2.99
GO Biological Process	GO:0030194	positive regulation of blood coagulation	7.2E-03	4.23	9.6E-02	2.70
GO Biological Process	GO:0072378	blood coagulation, fibrin clot formation	8.6E-03	4.05	6.3E-03	4.07
GO Biological Process	GO:0050820	positive regulation of coagulation	9.5E-03	3.95	1.2E-01	2.44
GO Biological Process	GO:0007597	blood coagulation, intrinsic pathway	1.2E-02	4.58	1.8E-02	3.80
PANTHER Pathway	P00011	Blood coagulation	3.0E-02	2.92	8.2E-02	2.14
MSigDB Pathway	REACTOME_FORMATION_OF_FIBRIN_CLOT_CLOTTING_CASCADE	Genes involved in Formation of Fibrin Clot (Clotting Cascade)	3.0E-02	2.92	4.1E-02	2.59
MSigDB Pathway	KEGG_COMPLEMENT_AND_COAGULATION_CASCADES	Complement and coagulation cascades	1E-01	1.90	5.8E-01	0.99

Shown are results of the region-based (binomial) and gene-based (hypergeometric) functional enrichment test for all coagulation and wound-healing related annotations tested using the GREAT method. Differentially methylated probes were used as input and show a consistent signal of enrichment for LTL-associated CpG sites.

LTL-DNAm correlation in subtelomeric regions

Here, subtelomeric regions included both ends of chromosomes. The ends were proportional (5% for each) to the length of the corresponding chromosome (Supplementary Figure 2).

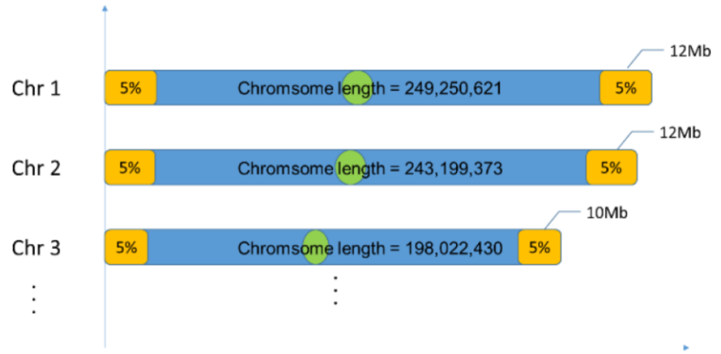

Supplementary Figure 2. Definition of subtelomeric regions.

We focused on the 823 significant CpG sites that were associated with the fully adjusted LTL. We counted the number of positive and negative Z-scores in non-subtelomeric and subtelomeric regions. The proportion of the positive LTL-DNAm correlations was 9.9% in non-subtelomeric bodies, whereas the corresponding proportion was 17.1% in subtelomeric regions.

Supplementary Table 3. Significant LTL-DNAm correlations by their sign (positive *vs* negative) and genomic location.

	Non-subtelomeric	Subtelomeric	Total
Negative	594	136	730
Positive	65	28	93
Total	659	164	823

Pearson's Chi-squared test with Yates' continuity correction.
χ-squared = 6.1099, df = 1, p-value = 0.01344

The chance of having significant CpGs was slightly higher in non-subtelomeric bodies than in subtelomeric regions (P=0.0427).

Supplementary Table 4. LTL-DNAm correlations by their significance and genomic location.

	Non-subtelomeric	Subtelomeric	Total
Non-significant (P>=1E-07)	339,775	101,272	441,047
Significant (P<1E-07)	659	164	823
Total	340,434	101,436	441,870

Pearson's Chi-squared test with Yates' continuity correction.
χ-squared = 4.1074, df = 1, p-value = 0.0427

Summary-data-based Mendelian randomization

Supplementary Table 5. Significant causal effects of 16 CpGs on LTL.

CpG	SNP	Gene	Chr	Global EWAS meta Z (P)	GWAS of LTL[1] beta (P)	mQTL[2] beta (P)	SMR[3] beta (P)	HEIDI[4] P-value
cg00622799	rs909334	RTEL1	20	-5.53 (3E-08)	0.04 (4E-05)	-0.23 (2E-10)	-0.17 (6E-04)	2.1E-01
cg19841423	rs2427533	ZGPAT;LIME1	20	-8.44 (3E-17)	0.03 (2E-03)	0.64 (4E-78)	0.04 (3E-03)	2.3E-06
cg04363228	rs2734335	PBX2	6	-6.22 (5E-10)	0.02 (2E-03)	0.29 (1E-18)	0.08 (3E-03)	9.5E-02
cg18909389	rs497309	CLIC1	6	-5.40 (7E-08)	-0.04 (3E-03)	-0.72 (3E-57)	0.05 (3E-03)	5.9E-03
cg27259408	rs3181049	FDX1L;RAVER1	19	-5.39 (7E-08)	0.03 (3E-03)	0.45 (7E-27)	0.07 (4E-03)	3.2E-01
cg23531049	rs17678767	MAPKBP1	15	-6.40 (2E-10)	0.02 (5E-03)	-0.42 (3E-36)	-0.05 (7E-03)	1.4E-01
cg03443360	rs642758	PTPRA	20	-5.37 (8E-08)	-0.02 (4E-03)	0.23 (2E-12)	-0.09 (7E-03)	5.2E-01
cg03609639	rs888208	NKX2-3	10	-5.47 (5E-08)	-0.02 (1E-02)	0.49 (6E-42)	-0.04 (2E-02)	4.7E-01
cg13754259	rs11190128	NKX2-3	10	-6.18 (6E-10)	-0.03 (2E-02)	0.29 (9E-16)	-0.10 (2E-02)	5.4E-01
cg00686926	rs6806847	GRK7	3	-5.40 (7E-08)	0.02 (2E-02)	0.78 (2E-111)	0.02 (2E-02)	1.4E-02
cg01289541	rs9880460	SLC7A14	3	5.66 (2E-08)	0.02 (3E-02)	0.25 (1E-14)	0.06 (3E-02)	1.1E-02
cg12054453	rs8078424	TMEM49	17	5.93 (3E-09)	0.02 (4E-02)	-0.57 (3E-55)	-0.03 (4E-02)	6.4E-01
cg16936953	rs8078424	TMEM49	17	5.63 (2E-08)	0.02 (4E-02)	-0.47 (2E-37)	-0.04 (4E-02)	8.3E-01
cg24531955	rs11777755	LOXL2	8	5.47 (5E-08)	-0.02 (4E-02)	-0.33 (2E-20)	0.05 (5E-02)	9.8E-01
cg21415060	rs3027077	FCER1A	1	-5.35 (9E-08)	-0.02 (3E-02)	-0.23 (2E-08)	0.09 (5E-02)	6.6E-01
cg14384960	rs13120596	ZNF827	4	-5.37 (8E-08)	-0.02 (4E-02)	0.18 (2E-08)	-0.09 (5E-02)	9.5E-01

[1]GWAS of LTL conducted by Codd and colleagues (2013), downloaded from https://downloads.lcbru.le.ac.uk/engage
[2]Methylation Quantitative trait locus (mQTL) provided by McRae and colleagues (2017), downloaded from http://cnsgenomics.com/data/SMR/LBC_BSGS_meta.tar.gz
[3]Summary-data-based Mendelian randomization (SMR) as proposed by Zhu and colleagues (2016).
[4]Heterogeneity in independent instruments (HEIDI) test.

Supplementary Table 6. Complex traits associated with the 22 cis-mQTL SNPs.

PMID	DISEASE/TRAIT	REGION	CHR	CHR_POS	REPORTED GENE(S)	SNPS	RISK ALLELE FREQUENCY	P-VALUE
28416818	Atrial fibrillation	2p14	2	65057097	CEP68	rs2540949	0.6100	3.00E-10
27863252	Neutrophil percentage of granulocytes	1p34.2	1	41905743	HIVEP3	rs2147904	0.5644	1.00E-09
27863252	Platelet count	19p13.12	19	16083694	TPM4	rs17708984	0.2879	6.00E-16
27863252	Platelet distribution width	19p13.12	19	16083694	TPM4	rs17708984	0.2882	2.00E-14
28448500	Waist circumference adjusted for body mass index	10q22.3	10	79147390	ZMIZ1	rs780159	0.5817	2.00E-06
28448500	Waist circumference adjusted for body mass index	10q22.3	10	79147390	ZMIZ1	rs780159	0.5817	6.00E-09
28448500	Waist circumference adjusted for body mass index	10q22.3	10	79147390	ZMIZ1	rs780159	0.5940	9.00E-09
28448500	Waist circumference adjusted for BMI in active individuals	10q22.3	10	79147390	ZMIZ1	rs780159	0.5817	3.00E-07
28448500	Waist circumference adjusted for BMI in active individuals	10q22.3	10	79147390	ZMIZ1	rs780159	0.5940	5.00E-07
28448500	Waist circumference adjusted for BMI	10q22.3	10	79147390	ZMIZ1	rs780159	0.5817	5.00E-08
28448500	Waist circumference adjusted for BMI	10q22.3	10	79147390	ZMIZ1	rs780159	0.5940	1.00E-07
27863252	Eosinophil counts	1p34.2	1	41905743	HIVEP3	rs2147904	0.5644	1.00E-14
27863252	Eosinophil percentage of granulocytes	1p34.2	1	41905743	HIVEP3	rs2147904	0.5644	2.00E-12
27863252	Sum eosinophil basophil counts	1p34.2	1	41905743	HIVEP3	rs2147904	0.5645	2.00E-12
27863252	Eosinophil percentage of white cells	1p34.2	1	41905743	HIVEP3	rs2147904	0.5644	2.00E-13
30061737	Atrial fibrillation	2p14	2	65057097	CEP68	rs2540949	0.6150	3.00E-22
29892015	Atrial fibrillation	2p14	2	65057097	CEP68	rs2540949	0.6200	8.00E-25
28604730	Lung cancer	6p21.33	6	31872700	SLC44A4	rs501942	0.0986	8.00E-19
28604730	Lung cancer in ever smokers	6p21.33	6	31872700	SLC44A4	rs501942	0.1010	3.00E-14
30595370	Eczema	1q25.1	1	173194429		rs7518129	NR	4.00E-18
30048462	Heel bone mineral density	4q31.22	4	145914638		rs6816078	NR	3.00E-18
30595370	Lung function (FEV1/FVC)	17q25.1	17	75523332		rs8064529	NR	1.00E-09

[1]GWAS catalogue database (v1.02) was downloaded from https://www.ebi.ac.uk/gwas/docs/file-downloads

Sensitivity analyses

Comparison of the biweight midcorrelation and ordinary linear regression

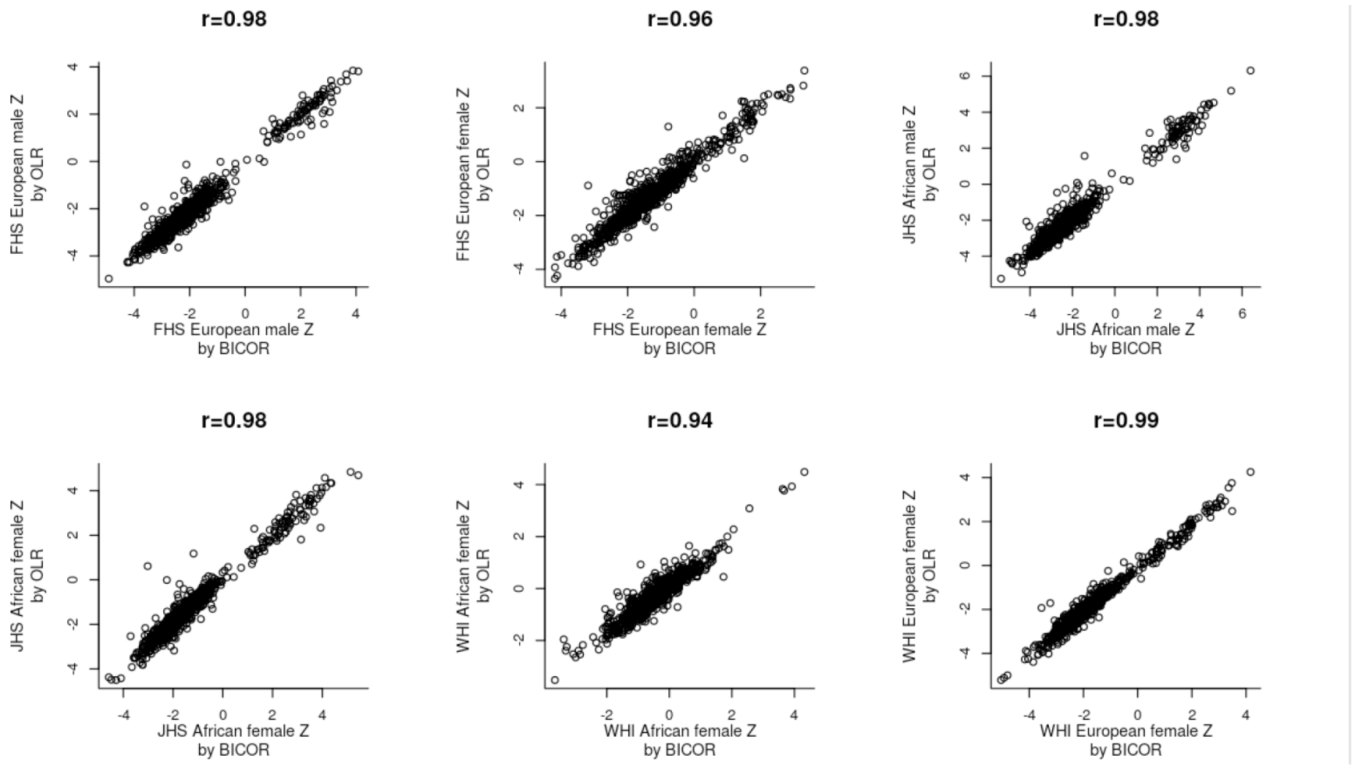

Supplementary Figure 3. Comparison of the BICOR and OLR method.

We conducted a sensitivity analysis to compare the biweight midcorrelation (BICOR) and ordinary linear regression (OLR) method for the EWAS of LTL. The panel above displays the Z scores generated by the BICOR and by the OLR method in the six strata (Supplementary Figure 3). The LTL was adjusted for age and the blood cell counts in each sex and ethnicity specific stratum as it was in the original analysis using the BICOR method. We replaced the BICOR with the OLR. For a clear presentation of results, we focused on the 823 significant CpG sites (fully adjusted) in this sensitivity analysis.

Additional adjustment for BMI (and education)

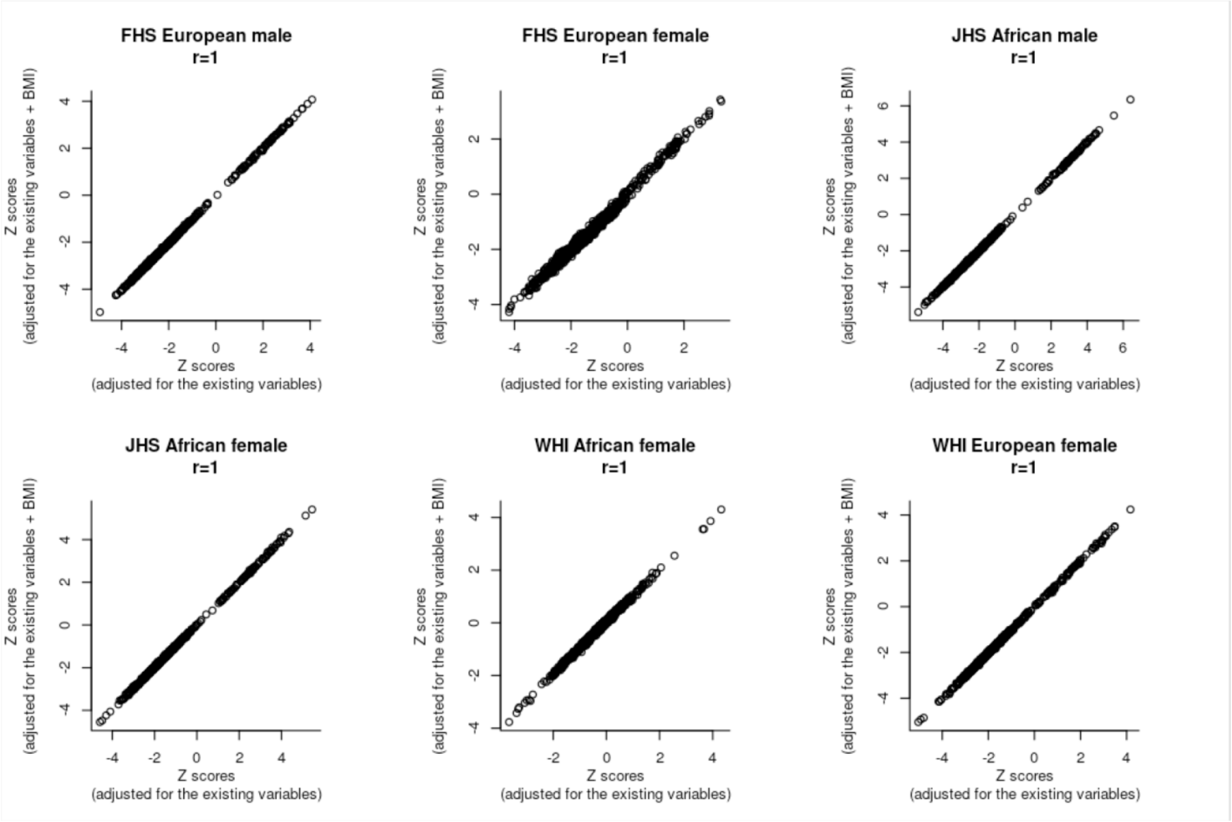

Supplementary Figure 4. Sensitivity analysis with or without additional adjustment for BMI.

We conducted a sensitivity analysis with and without additional adjustment for BMI using the three cohorts (FHS, JHS and WHI). Supplementary Figure 4 reveals that the Z scores adjusted for the existing variables (age, sex, ethnicity and blood cell counts) were almost same as the Z score adjusted for the existing variable and BMI. We did not observe any significant change.

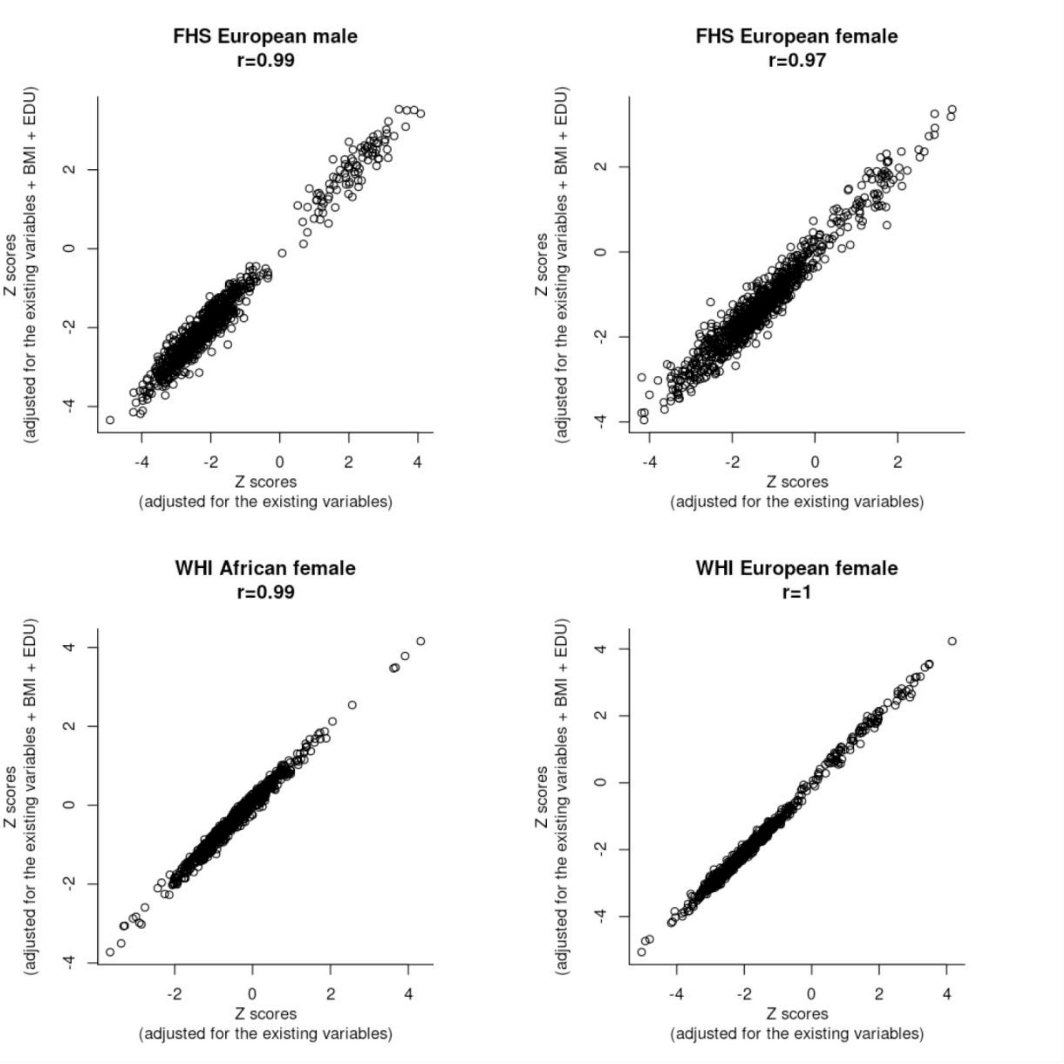

Supplementary Figure 5. Sensitivity analysis with or without additional adjustment for BMI and education.

We conducted another sensitivity analysis with and without additional adjustment for BMI and education using FHS and WHI. The education attainment of the JHS subjects was missing. Again, we did not observe any change by the additional adjustment for BMI and education.

Consistency across the cohorts (FHS, JHS, WHI, BHS, LBC21, LBC36 and LSADT)

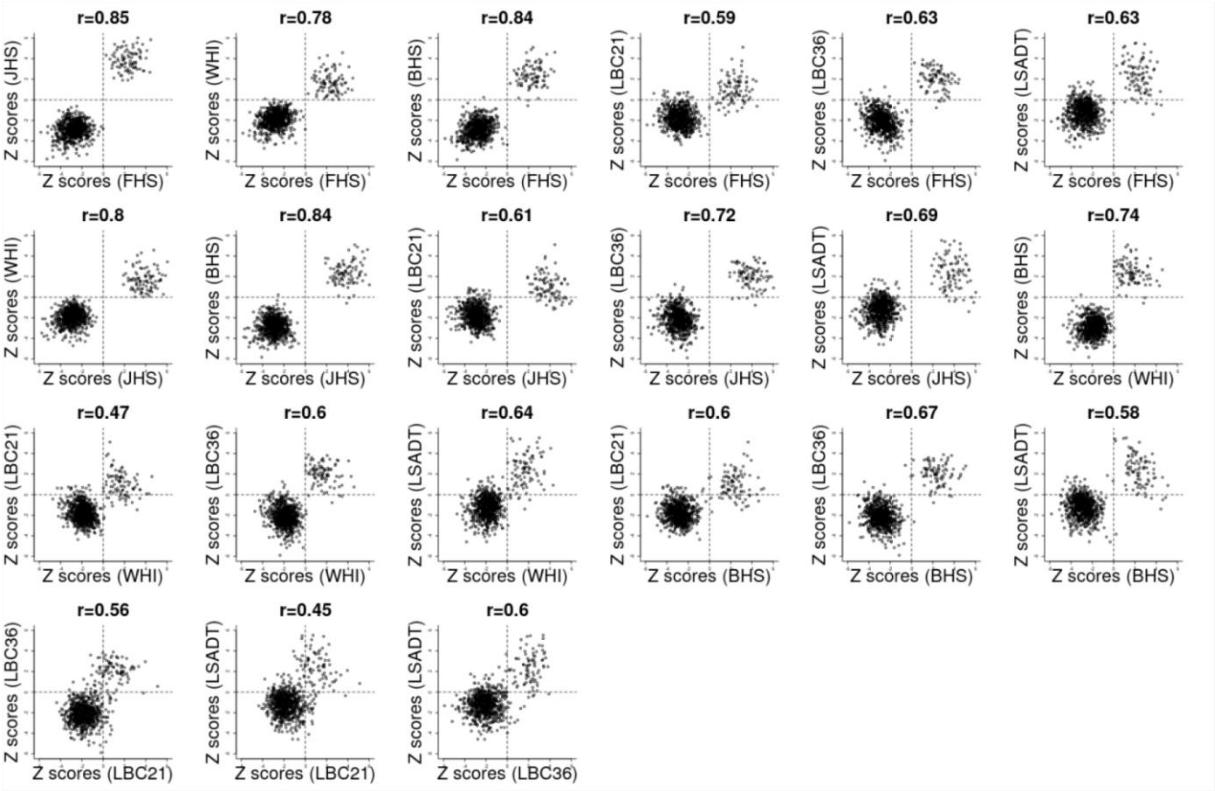

Supplementary Figure 6. Comparison the results across the seven cohorts.

We have also conducted a cohort-specific meta-analysis. We found the rough but consistent LTL-DNAm associations at the 823 CpG sites across the seven cohorts. Supplementary Figure 6 displays the Z scores from two cohorts using scatter plots (21 possible pairs/panels). The Stouffer's method ($\sum Z_i w_i / \sqrt{\sum w_i^2}$, where w_i is the square root of the sample size in the ith stratum) was used to combine corresponding strata for each cohort.

Study cohort

The Framingham Heart Study (FHS)

FHS started in 1948 to investigate common risk factors for cardiovascular disease (CVD) [2]. FHS recruited 5,209 subjects who lived in Framingham, Massachusetts, USA, and who were free from symptoms of CVD, heart attack or stroke at enrollment. The FHS Offspring Cohort started in 1971 and enrolled the original participants' grown-up children and the children's spouses (n=5,124) [3]. Our study included 874 participants from the FHS Offspring Cohort who attended the sixth and eighth examination and consented to the use of their bio-specimens for research purposes. Data from FHS can be retrieved from dbGaP (under accession numbers phs000363.v16.p10 and phs000724.v2.p9).

Telomere length measurement:

DNA was extracted from leukocytes collected from the sixth examination cycle. Participants who had enough buffy coat available were selected for LTL measurement. The Southern blot method was used to obtain the mean length of the terminal restriction fragment (TRF) as previously described [4]. The coefficient of variation was 2.4% for the LTL measurement of duplicate and triplicate DNA samples.

DNA methylation:

The Gentra Puregene DNA extraction kit (Qiagen) was used to extract genomic DNA from whole blood, which was then bisulfite-converted using the EZ DNA Methylation kit (Zymo Research Corporation). Data from FHS are accessible through dbGaP (accession numbers phs000363.v16.p10 and phs000724.v2.p9). FHS used the 'normal-exponential out-of-band' (noob, [5]) normalization method from the ***R*** package minfi [6].

The Jackson Heart Study (JHS)

JHS recruited 5,306 African Americans to investigate risk factors for cardiovascular disease in the Jackson metropolitan area, Mississippi, USA [7]. Participants provided medical and social records, physical and biochemical measurements, information on diagnostic procedures, and DNA samples during a baseline examination (2000-2004), and two follow-up examinations (2005-2008 and 2009-2012). JHS follows up the participants every year and maintains cohort surveillance. Our study included the participants who visited at the baseline examination as part of the ancillary study ASN0104 in JHS.

Telomere length measurement:

The Puregene kit (Gentra System, Minneapolis, MN, USA) [8] was used to extract DNA from whole blood. LTL (in kilobases) was measured using Southern blot [9]. The inter-assay coefficient of variation was 2.0%. The interclass correlation coefficient was 0.95 for individual measures of LTL.

DNA methylation:

DNA was extracted using the Gentra Puregene blood kit (Gentra System, MN, Minnesota, USA). JHS used the noob normalization method from the ***R*** package minfi [5, 10].

The Women's Health Initiative (WHI)

WHI started in 1992 and enrolled 64,500 postmenopausal women aged between 50 and 79 years into either clinical trials or observational studies [11]. Our study included WHI participants with available phenotype and DNA methylation array data referred to as the WHI "Broad Agency Award 23" (WHI BA23). WHI BA23 aimed to identify miRNA and genomic biomarkers of coronary heart disease.

Telomere length measurement:

DNA was extracted from blood samples collected at the time of the 2012–2013 visit, using the 5-prime method (5 PRIME, Inc.; Gaithersburg, MD). Prior to LTL measurement, DNA integrity was assessed visually after ethidium bromide-stained 1% agarose gel electrophoresis (200 V for 2 hours). The Southern blot method was used to measure the average length of the terminal restriction fragments (in kilobases) [12]. Individual samples were measured in duplicate on different gels. The average inter-assay coefficient of variation was 2.0%.

DNA methylation:

Among many sub-studies, only WHI BA23 provided both blood-based LTL and DNAm array data. WHI BA23 used the background correction method from Illumina's proprietary GenomeStudio software.

The Bogalusa Heart Study (BHS)

BHS started in 1972 and recruited multiple waves of participants from childhood, adolescent and adulthood in a biracial community in Bogalusa, Louisiana, USA, comprising 65% whites and 35% African Americans [13]. The longitudinal cardiovascular risk factor phenotype and genotype data of the BHS cohort are available via application through the NHLBI Biologic Specimen and Data Repository Information Coordinating Center website (https://biolincc.nhlbi.nih.gov/studies/bhs).

Telomere length measurement:

LTL was measured by Southern blot, as in the above studies. DNA was hybridized to a digoxigenin 3'-end labeled 5'-(CCCTAA)3 telomeric probe after overnight DNA digestion with 10 U Hinf I and 10 U Rsa I restriction enzymes as previously described [14]. Digitized autoradiograms of LTL measurement were analyzed for each sample resolved in duplicate on

different gels, and the coefficient of variation for the duplicate samples was 1.4% [14].

DNA methylation:

Genomic DNA was isolated from whole blood samples in BHS using the FlexiGene DNA extraction kit (Qiagen, Hilden, Germany). The Infinium HumanMethylation450K BeadChip (Illumina, San Diego, California, USA) was used for whole-genome DNAm analysis. Samples were processed at the Microarray Core Facility Lab, University of Texas Southwestern Medical Center, Dallas, Texas, USA. For each subject, 750 ng of genomic DNA was bisulfite-converted using the 96-well EZ DNAm kit (Zymo Research, Irvine, California, USA) according to the manufacturer's instructions. The efficiency of the bisulfite conversion was confirmed by in-built controls on the 450K array. The methylation profile of each participant was measured by processing 4 µl of bisulfite-converted DNA, at a concentration of 50 ng/µl, on an Illumina 450K array. The bisulfite-converted DNA was amplified, fragmented and hybridized to the array following the protocol. We scanned the arrays by using an Illumina iScan scanner, and then the raw methylation data was extracted using Illumina's GenomeStudio Methylation (M) Module. BHS used the data-driven separate normalization method (dasen, [15]) from the ***R*** package wateRmelon [15]. The probe exclusion criteria for filtering samples and probes were: 1) samples having 1% of CpG sites with a detection p-value greater than 0.05; 2) probes having 5% of samples with a detection p-value greater than 0.05; 3), and probes with bead count less than 3 in 5% of the samples.

The Lothian Birth Cohorts (LBC)

The Lothian Birth Cohorts of 1921 (LBC21) and 1936 (LBC36) are longitudinal studies of cognitive aging in individuals born in 1921 and 1936, respectively [16]. At age 11, these individuals had completed the Moray House Test of general intelligence as part of the Scottish Mental Surveys of 1932 and 1947. Decades later, individuals living in Edinburgh and the surrounding areas were contacted and invited to participate in wave 1 of the Lothian Birth Cohort (LBC) studies. Of those born in 1921, 550 individuals were recruited between 1999 and 2001 at mean age of 79. Of those born in 1936, 1091 individuals were recruited between 2004 and 2007 at mean age 70. Since then, extensive phenotypic data have been collected roughly every three years in four further waves of testing. The data collection includes detailed physical, cognitive, psychosocial and lifestyle measures. In addition, genetic and epigenetic data are available in both LBC21 and LBC36. More details on recruitment and testing can be found elsewhere [17, 18]. Our study here uses data obtained in the first wave of testing.

Telomere length measurement:

Telomere length in the LBC21 and LBC36 was measured in wave 1 using a quantitative real-time polymerase chain reaction (qPCR) assay [19]. DNA was extracted from whole blood at the Wellcome Trust Clinical Research Facility Genetics Core at the Western General Hospital in Edinburgh using standard procedures. A 7900HT Fast Real Time PCR machine with 384-well plate capacity (Applied Biosystems; Pleasanton, California, USA) was used to perform the PCRs. Telomere length was measured as the ratio of telomeric template to glyceraldehyde 3-phosphate dehydrogenase. Four internal control DNA samples derived from cell lines of known absolute telomere length were included on each plate to correct for plate-to-plate variation, and measurements were performed in quadruplicate and the mean was used in further assessments.

DNA methylation:

LBC used internal controls from the ***R*** package minfi to correct for background noise. Following this, samples of low quality, e.g. those with bisulfite conversion, staining signal, inadequate hybridization or nucleotide extension, were excluded. In addition, probes with a detection rate <95% at p<0.01 and samples with a low call rate (<450,000 probes detected at p<0.01) were removed. Finally, samples for which DNA-methylation predicted sex did not match reported sex and samples that showed a poor match between SNP control probes and genotype were removed.

The Longitudinal Study of Aging Danish Twins (LSADT)

LSADT was initiated in 1995 and recruited all Danish twins aged 70 years or more [9, 10]. Surviving twins were surveyed every other year until 2007. In 1997, whole-blood samples were collected from 689 same-sex twins. For 310 of these individuals, genome-wide DNAm data was available. The present study includes all twin pairs who participated in the 1997 wave and for whom genome-wide DNA methylation data and LTL measurements were available.

Telomere length measurement:

LTL was measured using Southern blot. The LTL measurement referred to the average terminal restriction fragments after digestion with HinfI and RsaI restriction enzymes as previously described [9]. The two LTL measures presented a high correlation (r=0.88, p=0.000). Each sample was used in duplicate on different gels. The inter-assay coefficient of variation was 2.5% (for the HinfI/RsaI digest).

DNA methylation:

The EZ Methylation Gold kit (Zymo Research, Orange County, California, USA) was used to isolate DNA

from buffy coats. LSADT used the functional normalization method [10] from the ***R*** package minfi for normalization of the methylation data. The following criteria were used for sample exclusion: firstly, samples where less than 95% of the probes had a detection P-value < 0.01, and secondly, samples that failed inspection of the internal quality control probes of the bead chip, which is done using the ***R***/Bioconductor package MethylAid for identifying low-quality samples (see van Iterson et al. (2014) for details, [20]). Probes were excluded if they satisfied at least one of the following criteria: a detection P-value >0.01, a raw intensity value of zero, a low bead count (< 3 beads), were identified as being cross reactive [21], and/or and had a measurement success rate below 95%. After probe filtering, the criterion of 95% sample success rate was applied to the remaining data none of the samples had a lower sample success rate and therefore none of them was excluded at this step (see Debrabant et al. (2018) for further details regarding the LSADT DNA methylation data [22]).

REFERENCES

1. McLean CY, Bristor D, Hiller M, Clarke SL, Schaar BT, Lowe CB, Wenger AM, Bejerano G. GREAT improves functional interpretation of cis-regulatory regions. Nat Biotechnol. 2010; 28:495–501. https://doi.org/10.1038/nbt.1630 PMID:20436461

2. Dawber TR, Meadors GF, Moore FE Jr. Epidemiological approaches to heart disease: the Framingham Study. Am J Public Health Nations Health. 1951; 41:279–81. https://doi.org/10.2105/AJPH.41.3.279 PMID:14819398

3. Kannel WB, Feinleib M, McNamara PM, Garrison RJ, Castelli WP. An investigation of coronary heart disease in families. The Framingham offspring study. Am J Epidemiol. 1979; 110:281–90. https://doi.org/10.1093/oxfordjournals.aje.a112813 PMID:474565

4. Huzen J, van Veldhuisen DJ, van der Harst P. Letter by Huzen et al regarding article, "Association of leukocyte telomere length with circulating biomarkers of the renin-angiotensin-aldosterone system: the Framingham Heart Study". Circulation. 2008; 118:e688. https://doi.org/10.1161/CIRCULATIONAHA.108.775510 PMID:18981308

5. Triche TJ Jr, Weisenberger DJ, Van Den Berg D, Laird PW, Siegmund KD. Low-level processing of Illumina Infinium DNA Methylation BeadArrays. Nucleic Acids Res. 2013; 41:e90. https://doi.org/10.1093/nar/gkt090 PMID:23476028

6. Aryee MJ, Jaffe AE, Corrada-Bravo H, Ladd-Acosta C, Feinberg AP, Hansen KD, Irizarry RA. Minfi: a flexible and comprehensive Bioconductor package for the analysis of Infinium DNA methylation microarrays. Bioinformatics. 2014; 30:1363–69. https://doi.org/10.1093/bioinformatics/btu049 PMID:24478339

7. Taylor HA Jr, Wilson JG, Jones DW, Sarpong DF, Srinivasan A, Garrison RJ, Nelson C, Wyatt SB. Toward resolution of cardiovascular health disparities in African Americans: design and methods of the Jackson Heart Study. Ethn Dis. 2005 Autumn;15(4 Suppl 6):S6-4-17. PMID:16320381

8. Carpenter MA, Crow R, Steffes M, Rock W, Heilbraun J, Evans G, Skelton T, Jensen R, Sarpong D. Laboratory, reading center, and coordinating center data management methods in the Jackson Heart Study. Am J Med Sci. 2004; 328:131–44. https://doi.org/10.1097/00000441-200409000-00001 PMID:15367870

9. Kimura M, Stone RC, Hunt SC, Skurnick J, Lu X, Cao X, Harley CB, Aviv A. Measurement of telomere length by the Southern blot analysis of terminal restriction fragment lengths. Nat Protoc. 2010; 5:1596–607. https://doi.org/10.1038/nprot.2010.124 PMID:21085125

10. Fortin JP, Triche TJ Jr, Hansen KD. Preprocessing, normalization and integration of the Illumina HumanMethylationEPIC array with minfi. Bioinformatics. 2017; 33:558–60. PMID:28035024

11. Anderson G, et al, and The Women's Health Initiative Study Group. Design of the Women's Health Initiative clinical trial and observational study. Control Clin Trials. 1998; 19:61–109. https://doi.org/10.1016/S0197-2456(97)00078-0 PMID:9492970

12. Loprinzi PD, Loenneke JP, Blackburn EH. Movement-Based Behaviors and Leukocyte Telomere Length among US Adults. Med Sci Sports Exerc. 2015; 47:2347–52. https://doi.org/10.1249/MSS.0000000000000695 PMID:25970659

13. Berenson GS, Co-Investigators BH. Bogalusa Heart Study: a long-term community study of a rural biracial (black/white) population. Am J Med Sci. 2001; 322:267–74. https://doi.org/10.1097/00000441-200111000-00007 PMID:11721800

14. Aviv A, Chen W, Gardner JP, Kimura M, Brimacombe M, Cao X, Srinivasan SR, Berenson GS. Leukocyte telomere dynamics: longitudinal findings among young adults in the Bogalusa Heart Study. Am J

Epidemiol. 2009; 169:323–29. https://doi.org/10.1093/aje/kwn338 PMID:19056834

15. Pidsley R, Y Wong CC, Volta M, Lunnon K, Mill J, Schalkwyk LC. A data-driven approach to preprocessing Illumina 450K methylation array data. BMC Genomics. 2013; 14:293. https://doi.org/10.1186/1471-2164-14-293 PMID:23631413

16. Deary IJ, Gow AJ, Pattie A, Starr JM. Cohort profile: the Lothian Birth Cohorts of 1921 and 1936. Int J Epidemiol. 2012; 41:1576–84. https://doi.org/10.1093/ije/dyr197 PMID:22253310

17. Shah S, McRae AF, Marioni RE, Harris SE, Gibson J, Henders AK, Redmond P, Cox SR, Pattie A, Corley J, Murphy L, Martin NG, Montgomery GW, et al. Genetic and environmental exposures constrain epigenetic drift over the human life course. Genome Res. 2014; 24:1725–33. https://doi.org/10.1101/gr.176933.114 PMID:25249537

18. Marioni RE, Shah S, McRae AF, Chen BH, Colicino E, Harris SE, Gibson J, Henders AK, Redmond P, Cox SR, Pattie A, Corley J, Murphy L, et al. DNA methylation age of blood predicts all-cause mortality in later life. Genome Biol. 2015; 16:25. https://doi.org/10.1186/s13059-015-0584-6 PMID:25633388

19. Martin-Ruiz C, Saretzki G, Petrie J, Ladhoff J, Jeyapalan J, Wei W, Sedivy J, von Zglinicki T. Stochastic variation in telomere shortening rate causes heterogeneity of human fibroblast replicative life span. J Biol Chem. 2004; 279:17826–33. https://doi.org/10.1074/jbc.M311980200 PMID:14963037

20. van Iterson M, Tobi EW, Slieker RC, den Hollander W, Luijk R, Slagboom PE, Heijmans BT. MethylAid: visual and interactive quality control of large Illumina 450k datasets. Bioinformatics. 2014; 30:3435–37. https://doi.org/10.1093/bioinformatics/btu566 PMID:25147358

21. Chen YA, Lemire M, Choufani S, Butcher DT, Grafodatskaya D, Zanke BW, Gallinger S, Hudson TJ, Weksberg R. Discovery of cross-reactive probes and polymorphic CpGs in the Illumina Infinium HumanMethylation450 microarray. Epigenetics. 2013; 8:203–09. https://doi.org/10.4161/epi.23470 PMID:23314698

22. Debrabant B, Soerensen M, Christiansen L, Tan Q, McGue M, Christensen K, Hjelmborg J. DNA methylation age and perceived age in elderly Danish twins. Mech Ageing Dev. 2018; 169:40–44. https://doi.org/10.1016/j.mad.2017.09.004 PMID:28965790
